# Analysis of conservation priorities of Iberoamerican cattle based on autosomal microsatellite markers

**DOI:** 10.1186/1297-9686-45-35

**Published:** 2013-09-30

**Authors:** Catarina Ginja, Luís T Gama, Óscar Cortes, Juan Vicente Delgado, Susana Dunner, David García, Vincenzo Landi, Inmaculada Martín-Burriel, Amparo Martínez-Martínez, M Cecília T Penedo, Clementina Rodellar, Pilar Zaragoza, Javier Cañon

**Affiliations:** 1 Centro de Biologia Ambiental, Faculdade de Ciências, Universidade de Lisboa, Lisboa, Portugal; 2 CIISA, Faculdade de Medicina Veterinária, Universidade Técnica de Lisboa, Lisboa, Portugal; 3 Departamento de Producción Animal, Facultad de Veterinaria, Universidad Complutense de Madrid, Madrid, Spain; 4 Departamento de Genética, Universidad de Córdoba, Córdoba, Spain; 5 Laboratorio de Genética Bioquímica, Facultad de Veterinaria, Universidad de Zaragoza, Zaragoza, Spain; 6 Veterinary Genetics Laboratory, University of California, Davis, California, USA

## Abstract

**Background:**

Determining the value of livestock breeds is essential to define conservation priorities, manage genetic diversity and allocate funds. Within- and between-breed genetic diversity need to be assessed to preserve the highest intra-specific variability. Information on genetic diversity and risk status is still lacking for many Creole cattle breeds from the Americas, despite their distinct evolutionary trajectories and adaptation to extreme environmental conditions.

**Methods:**

A comprehensive genetic analysis of 67 Iberoamerican cattle breeds was carried out with 19 FAO-recommended microsatellites to assess conservation priorities. Contributions to global diversity were investigated using alternative methods, with different weights given to the within- and between-breed components of genetic diversity. Information on Iberoamerican plus 15 worldwide cattle breeds was used to investigate the contribution of geographical breed groups to global genetic diversity.

**Results:**

Overall, Creole cattle breeds showed a high level of genetic diversity with the highest level found in breeds admixed with zebu cattle, which were clearly differentiated from all other breeds. Within-breed kinships revealed seven highly inbred Creole breeds for which measures are needed to avoid further genetic erosion. However, if contribution to heterozygosity was the only criterion considered, some of these breeds had the lowest priority for conservation decisions. The Weitzman approach prioritized highly differentiated breeds, such as Guabalá, Romosinuano, Cr. Patagonico, Siboney and Caracú, while kinship-based methods prioritized mainly zebu-related breeds. With the combined approaches, breed ranking depended on the weights given to the within- and between-breed components of diversity. Overall, the Creole groups of breeds were generally assigned a higher priority for conservation than the European groups of breeds.

**Conclusions:**

Conservation priorities differed significantly according to the weight given to within- and between-breed genetic diversity. Thus, when establishing conservation programs, it is necessary to also take into account other features. Creole cattle and local isolated breeds retain a high level of genetic diversity. The development of sustainable breeding and crossbreeding programs for Creole breeds, and the added value resulting from their products should be taken into consideration to ensure their long-term survival.

## Background

Today, it is estimated that throughout the world, there is one cow for every five people. The most recent report of the Food and Agriculture Organization (FAO) on farm animal genetic resources [1] illustrates the importance of cattle as a domestic species. Overall, cattle breeds represent about 22% of the documented mammalian livestock breeds, but among these, 16% are extinct, 16% are threatened and for 30% information on population sizes and number of breeding animals is lacking. This erosion in cattle diversity occurred mainly during the last century for different reasons i.e. commercial breeds were preferred to native populations, crossbreeding led to dilution, and the widespread use of artificial insemination resulted in a sharp decline in sex ratio [2,3].

The risk status of each breed is defined on the basis of both demographic and cultural aspects [4]. For example, the FAO has developed a public database [5] as a warning system for worldwide domestic animal genetic resources (AnGR), in which breeds with less than ~1000 breeding females and/or less than ~20 breeding males are classified as threatened [6]. As a preservation measure, the European Union provides financial support to livestock producers of native breeds that are considered in risk of abandonment [2]. Nonetheless, there is general agreement that assessment and preservation of genetic diversity in livestock species are fundamental to meet future breeding needs, to investigate the genetic basis of phenotypic variation and to reconstruct the history of farm animals [7,8]. Autosomal microsatellites have been the most used genetic markers to estimate diversity parameters, to investigate breed relationships and to define conservation priorities [8]. Preservation of 'neutral’ genetic diversity is expected to contribute to maintaining specific breed traits due to natural and artificial selection. Indeed, microsatellites can be present in genes associated with important traits, including adaptation [9,10].

Determining the conservation value of breeds is essential to define priorities, including allocation of funds, and to manage extant genetic diversity. Several methods based on the establishment of conservation priorities have been developed to maintain high levels of neutral genetic variation [11,12]. The Weitzman approach [13] has been widely used in conservation analyses of domestic animal populations (for a review see [12]), including cattle [14-18]. This method uses a matrix of genetic distances as a measure of between-breed diversity and thus puts conservation priorities on the breeds that are more differentiated, i.e. that cause the greatest reduction in branch lengths of a phylogenetic tree when they are removed from the metapopulation. Weitzman [13] introduced the concept of marginal diversity to account for the change in expected diversity of a metapopulation (for a given period of time) when the extinction probability of a sub-population increases [12]. Thus, in the Weitzman approach genetic diversity is maximized if genetically distant breeds (i.e. more unique) are preserved. However, this method has been strongly criticized because it ignores within-breed diversity [11,17,19-23]. Moreover, breed uniqueness inferred from genetic distances may result from prevalence of rare alleles due to inbreeding, founder effects or strict genetic isolation, instead of a distinct evolutionary history [10,17].

Notwithstanding these limitations, it is important to consider that the genetic diversity of a livestock species results from the genetic variation both within- and between-breeds in order to preserve the highest intra-specific variability [8,12,24]. Within-breed diversity is routinely quantified by Nei’s [25] expected heterozygosity, but alternative methods based on allelic diversity can also be used [26,27]. Strategies that maximize heterozygosity keep the levels of allelic diversity as high as strategies that maximize allelic diversity, while they minimize the levels of inbreeding [11]. Thus, when prioritizing breeds for conservation, deciding whether genetic or allelic diversity should be considered depends on whether the focus is on short-term (e.g. avoiding inbreeding in highly threatened breeds) or long-term (e.g. future adaptation to changing environments) goals, or on an optimal combination of both (for a discussion see [27]). In practice, the contribution of each breed to global diversity could be simply estimated as the percentage of gene and/or allelic diversity lost or gained by its removal (as in an extinction scenario) from the metapopulation considered [28]. However, this would fail to give proper weight to the between-breed component, including the possibility of calculating negative estimates for certain breeds.

Given the limitations of the above methods, alternative approaches have been proposed for breed prioritization, which aim at minimizing global molecular coancestry (i.e. inbreeding) considering both within- and between-breed kinship coefficients [20,23,29,30]. For native European cattle breeds, methods based on molecular kinships have been used to assess their value for conservation decisions [9,16-18,31,32]. Although genealogical inbreeding is a good predictor of molecular inbreeding, the opposite might not be true and depends highly on the number of genetic markers used to estimate heterozygosity [11]. Thus, the ideal situation would be to use a combination of pedigree and molecular approaches, but in many cases reliable genealogical data is unavailable. For management purposes, extensions of these methods have been proposed in which acceptable rates of inbreeding and exchange between breeds are defined [27,33].

Assuming that both within- and between-breed contributions to genetic diversity should be taken into account to define conservation priorities, various weights applied to each component have been proposed [20,34-37]. For example, Ollivier and Foulley [37] used the Weitzman estimate of between-breed diversity weighted by the overall degree of population differentiation, i.e. Wright’s fixation index F_ST_[38], combined with the within-breed diversity (measured as the proportional loss in global heterozygosity when a breed is 'lost’) weighted by 1-F_ST_. It has been argued that higher weights should be given to the between-breed dimension [34] or that the total genetic variance of a hypothetical trait should be considered [36], but no consensus has been reached on a single method. Cañon et al. [39] carried out a comparative analysis to assess conservation priorities in a comprehensive study of Iberian native cattle, using several of the above-mentioned methods. While their study did not make any definite recommendation, it provided a wide perspective on how genetic variation is distributed among peripheral and more variable breeds, which can be helpful for decision-makers to carry out conservation programs.

Several studies have aimed at characterizing the genetic diversity of worldwide cattle (for a review see [7]). However, in many cases different marker panels were used and the number of breeds was limited, thus a large-scale comparison of the results, both in terms of genetic diversity and conservation priorities, was difficult. Europe and the Americas, including both North and Latin America, hold about 47% of the worldwide cattle population [1]. In Europe, local breeds are recognized as important AnGR and information on their genetic diversity and risk status is generally available. However, for many populations of Creole cattle from Latin America, such information essential for conservation purposes is lacking, in spite of their distinct evolutionary trajectories and adaptation to extreme environmental conditions. Previously, we reported data on the genetic diversity and breed structure of Creole cattle using FAO-recommended microsatellite markers [40], based on a large dataset of Iberoamerican cattle and other European and Indicine breeds. In the present study, we conducted a comprehensive analysis of conservation priorities of Creole cattle, with the aim of assessing their value for conservation decisions and management of animal genetic resources. Overall, data on more than 80 worldwide bovine breeds was used to investigate the contribution of different geographical breed groups to the global genetic diversity of cattle.

## Methods

### Sampling

We analyzed 3383 animals belonging to 82 populations of Iberoamerican cattle that included British, Continental European and Indicine breeds from 12 countries. Animals were sampled within the framework of the BIOBOVIS consortium [41] according to recommended procedures for the collection of biological specimens (blood, semen or hair roots) from cattle. Details on the sampling procedures and breed distributions are reported elsewhere [40,42-44]. International and country regulations regarding experimental research on animals were strictly followed.

Based on previous results [40,43,44], relationships between cattle breeds were used to establish 13 major breed groups [for details see Additional file 1: Table S1]. Conservation analyses based on geographical distributions and distinct breed types (e.g. taurine, indicine and crossbred) were performed. Specifically, Creole cattle of the Americas (907 animals; 27 breeds) were distributed into six major groups: Creole1 (175 animals; 4 breeds), cattle from the southern region of South America that are related to Iberian breeds and partly to African cattle; Creole2 (121; 4), cattle populations from Colombia and Paraguay admixed with British breeds; Creole3 (50; 2), Colombian cattle related to Iberian breeds; Creole4 (61; 2), cattle from Panama, possibly with some influence from African zebu; Creole5 (212; 6), mostly Mexican cattle admixed with Continental European breeds and also with zebu; and Creole6 (288; 9), cattle from central America extensively admixed with zebu. Iberian breeds were separated into four groups: Iberian1 (609; 12), most of the Portuguese breeds; Iberian2 (452; 10), Spanish and Portuguese breeds that are known to have been admixed with commercial European breeds; Iberian3 (100; 2), breeds from the Canary Islands; and Iberian4 (763; 16), most of the Spanish breeds. For comparison purposes, we also analyzed other breeds that are known to have influenced the Creole cattle of the Americas and that were classified into three groups: British (200; 5), includes mainly commercial breeds of British origin; Continental Europe (184; 4), commercial breeds that originate from Continental Europe; and Indicine (*Bos indicus,* 168; 6), widely spread zebu breeds.

### Microsatellite genotyping

We used a microsatellite dataset previously generated by the BIOBOVIS research consortium [40], which is available to the scientific community from a public database [45]. All animals were genotyped with a panel of 19 microsatellite loci: *BM1818*, *BM1824*, *BM2113*, *CSRM060*, *CSSM066*, *ETH003*, *ETH010*, *ETH185*, *ETH225*, *HAUT027*, *HEL009*, *ILSTS006*, *INRA032*, *INRA063*, *MM12E6*, *SPS115*, *TGLA053*, *TGLA122*, and *TGLA227*. These loci are distributed across 17 cattle autosomes and are recommended by the International Society for Animal Genetics (ISAG) / Food and Agriculture Organization of the United Nations (FAO) Advisory Committee for genetic diversity studies. The genotyping and allele standardization procedures have been validated and are described in detail in [40].

### Statistical analyses

Estimates of within-breed genetic diversity were obtained with GENETIX 4.05.2 software [46], namely observed (H_o_) and unbiased expected (H_e_) heterozygosities, mean number of alleles (MNA) per breed and geographical breed group. This software was also used to estimate *F* statistics according to Weir and Cockerham [38]. Allelic richness (R_t_) over all loci per breed was calculated with FSTAT v. 2.9.3 software [47].

Analysis of conservation priorities depends on how the metapopulation is defined to investigate partial contributions to global genetic diversity. Also, breed prioritization will vary considerably according to the relative importance of the within- and between-breed components of genetic diversity in each breed and to the genetic relationships among breeds. Given the extensive collection of cattle specimens available for this study and for the sake of simplicity, we took into consideration two dimensions that were analyzed separately i.e., (1) the 27 Creole cattle populations and (2) the 13 major geographical breed groups defined above. From the perspective of practical applications, we chose to consider geographical breed groups with their specificities, rather than the complete worldwide distribution of cattle included in a single metapopulation.

For the conservation analyses, we followed the methods described by Cañon et al. [39] and outlined below. For a better understanding of the statistical procedures involved, we categorized the different approaches as follows: methods that aim at minimizing the overall kinship coefficient of the metapopulation (kinship-based methods), a method that reflects only the between-breed diversity component (Weitzman approach) and combined approaches that take into consideration both the within- and between-breed components of global genetic diversity.

### Kinship-based methods

We applied the Core Set methods of Eding et al. [21] to investigate the population contributions to global diversity that account for within- and between-breed kinship coefficients by minimizing the overall kinship coefficient of the metapopulation considered and eliminating the genetic overlap between breeds in the core set [12]. Estimation of possible negative contributions by a given population is avoided through an iterative process that gives the lowest value a zero and recalculates the contributions after removal of the population.

In the absence of genealogical data, kinships were estimated from molecular data with different methods: (1) marker-estimated kinships (MEK) obtained from individual genotypes, as described by Eding and Meuwissen [29]; (2) a variation of the MEK method based on log-linear regressions [30] obtained with the weighted log-linear model (WLM); (3) same as (2) but the log-linear regressions were obtained with the mixed model (WLMM); and (4) average molecular coancestries (*f*_m_) based on allele frequencies [20]. MEK were estimated with a macro function in Excel [39], whereas the solutions for WLM and WLMM were obtained with matrices built with the MATLAB® software (The MathWorks, Inc., USA). Average coancestry coefficients within (*f*_ii_) and between (*f*_ij_) each Creole breed and geographical breed group were calculated with the MOLKIN3 software [48]. Analyses of conservation priorities based on these similarity matrices (MEK, WLM, WLMM and *f*_m_) were carried-out with a FORTRAN program developed and kindly shared by Eding and Meuwissen.

We derived pairwise kinship distances from the MEK coefficients following Eding et al. [21] as: d(_i, j_) = *f*_ii_ + *f*_jj_ - 2*f*_ij_. Kinship genetic distances were used to construct the neighbor-net phylogenies of the Creole breeds and geographical breed groups with the SPLITS TREE4 4.12.6 software [49]. Genetic relationships were used to classify the Creole breeds and groups of breeds- and then to build contour plots of kinship coefficients (MEK and *f*_m_) with the MATLAB® software (The MathWorks, Inc., USA).

In order to assess within-breed genetic diversity directly, the partial contributions of each Creole breed and each geographical breed group were also calculated as the proportional variation in expected heterozygosity of the metapopulation after removal of each breed or breed group (PC_He_).

### Weitzman approach

We calculated the partial contributions (PC_Weitz_) of each Creole breed and each geographical breed group to the total genetic diversity using the Weitzman method [13]. Here, Reynolds genetic distances [50] were used as a measure of between-breed diversity, while within-breed diversity was ignored. This approach estimates the reduction in length of the branches in a maximum likelihood phylogeny after removal of a population [20]. PC_Weitz_ were calculated with the FORTRAN program developed by Garcia et al. [22]. An alternative approximation algorithm developed by Garcia et al. [22] was also used to analyze the 13 geographical breed groups. Thresholds ranging from 0.550 to 0.001 were tested to verify the coherence of the results. Pairwise Reynolds genetic distances were calculated with the POPULATIONS 1.2.32 software [51] and used to obtain neighbor-net phylogenies of the Creole breeds and geographical breed groups built with the SPLITS TREE4 4.12.6 software [49].

### Combined approaches

Ideally, analyses of conservation priorities should take into account both within- and between-population genetic variability in order to make more accurate management decisions. We used three approaches to calculate contributions that combine these two levels of the global diversity of the metapopulation: (1) aggregate diversity (PC_Fst_) [37], which uses Wright’s F_st_ to weight the between- and (1-F_st_) to weigh the within-population components of diversity i.e., PC_Fst_ = PC_Weitz_*F_st_ + PC_He_*(1-F_st_); (2) the approach of Piyasatian and Kinghorn [34], which gives a weight five times higher to the between-population component (PC_5:1_), such that PC_5:1_ = PC_Weitz_*0.833 + PC_He_*(1–0.833); and (3) the method proposed by Caballero and Toro [20] and Fabuel et al. [35], which gives equal weights to within-population coancestries and genetic distances. In this case, Nei’s minimum distances [52] were used and calculations were done with MOLKIN3 software [48].

## Results

Results are organized according to the two dimensions defined above i.e., the 27 Creole cattle populations and the 13 geographical breed groups [see Additional file 1: Table S1].

### Within-breed diversity and breed relationships

Summary statistics that describe the genetic diversity within the Creole cattle breeds and the geographical breed groups are in Tables 1 and 2, respectively. With a few exceptions, Creole breeds had a high level of genetic diversity (on average, H_o_ = 0.719±0.053, H_e_ = 0.739±0.049, MNA = 6.95±1.52 and R_t_ = 4.71±1.1). Among the Creole cattle, the Creole1 and Creole3 groups of breeds that are related to Iberian or British breeds and also to African cattle, showed intermediate to high levels of genetic diversity (on average, H_o_ > 0.671±0.123 and H_e_ > 0.708±0.116), while the Creole breeds admixed with zebu cattle (Creole5 and Creole6) had high values across all estimates (on average, H_o_ > 0.712±0.091, H_e_ > 0.777±0.079, MNA > 10.84±2.34 and R_t_ > 4.92±1.17).

**Table 1:** Within-breed genetic diversity of Creole (Cr) cattle breeds

**Group**	**Country**	**Breed name**	**Acron**	**FAO risk status**	**N**	**H**_**o**_**±SD**	**H**_**e**_**±SD**	**MNA±SD**	**R**_**t**_**±SD**
Cr 1	Argentina	Cr Argentino	CRA	Unknown	50	0.673±0.110	0.678±0.101	6.26±1.66	4.00±0.80
Cr 1	Argentina	Cr Patagonico	PAT	Unknown	35	0.629±0.124	0.670±0.108	5.32±1.57	3.84±0.84
Cr 1	Brazil	Caracú	CAR	Not at risk	47	0.733±0.101	0.711±0.095	6.74±1.73	4.32±0.81
Cr 1	Uruguay	Cr Uruguayo	CRU	Endangered maintained	43	0.668±0.107	0.674±0.085	5.63±1.67	3.97±0.78
Cr 2	Colombia	Blanco Orejinegro	BON	Not at risk	25	0.737±0.127	0.697±0.100	5.74±1.76	4.10±0.82
Cr 2	Colombia	Hartón del Valle	HVA	Not at risk	22	0.783±0.130	0.783±0.070	7.74±1.73	5.24±0.92
Cr 2	Colombia	Lucerna	LUC	Not at risk	24	0.673±0.152	0.717±0.108	6.63±2.06	4.69±1.11
Cr 2	Paraguay	Pampa Chaqueño	PCH	Endangered	50	0.750±0.091	0.771±0.074	8.11±1.79	5.05±0.92
Cr 3	Colombia	Costeño con Cuernos	CCC	Endangered maintained	25	0.692±0.168	0.671±0.135	5.26±1.37	3.94±0.95
Cr 3	Colombia	Romosinuaño	RMS	Not at risk	25	0.651±0.140	0.669±0.132	5.11±1.59	3.94±1.00
Cr 4	Panama	Guabalá	GUA	Not reported	25	0.629±0.218	0.660±0.196	5.79±1.96	4.10±1.30
Cr 4	Panama	Guaymí	GYM	Not reported	36	0.735±0.082	0.756±0.075	7.79±1.65	4.93±0.91
Cr 5	Colombia	Sanmartinero	SMA	Not at risk	25	0.692±0.124	0.721±0.079	6.37±1.16	4.39±0.73
Cr 5	Mexico	Cr Baja California	CBC	Not at risk	21	0.742±0.158	0.760±0.082	7.05±1.58	4.98±0.98
Cr 5	Mexico	Cr Chihuahua	CHU	Unknown	19	0.719±0.168	0.777±0.080	6.68±1.49	5.14±0.95
Cr 5	Mexico	Cr Nayarit	CNY	Not at risk	24	0.749±0.121	0.788±0.077	7.74±1.94	5.25±0.89
Cr 5	Mexico	Cr Poblano	CPO	Unknown	43	0.693±0.108	0.774±0.076	8.37±2.01	5.07±1.01
Cr 5	USA	Texas Longhorn	TLH	Not at risk	80	0.707±0.117	0.740±0.111	8.05±2.46	4.78±1.13
Cr 6	Colombia	Caqueteño	CAQ	Critical	25	0.780±0.147	0.787±0.075	7.58±1.57	5.22±0.97
Cr 6	Colombia	Chino Santandereano	CHS	Unknown	25	0.726±0.091	0.776±0.055	7.32±1.73	5.03±0.80
Cr 6	Colombia	Cr Casanareño	CAS	Not at risk	35	0.739±0.414	0.766±0.078	8.00±1.65	*n*
Cr 6	Colombia	Velasquez	VEL	Unknown	25	0.730±0.122	0.769±0.069	6.79±1.44	4.92±0.87
Cr 6	Cuba	Cr Cubano	CUB	Not at risk	50	0.793±0.123	0.761±0.080	7.58±2.36	4.92±1.17
Cr 6	Cuba	Siboney	SIB	Unknown	50	0.746±0.172	0.762±0.116	8.05±2.30	5.05±1.08
Cr 6	Ecuador	Cr Ecuatoriano	ECU	Not at risk	12	0.732±0.174	0.772±0.100	6.63±2.11	5.23±1.22
Cr 6	Mexico	Cr Chiapas	CHI	Unknown	30	0.741±0.145	0.782±0.091	7.84±1.57	5.23±0.89
Cr 6	Paraguay	Pilcomayo	PIL	Unknown	36	0.764±0.125	0.769±0.096	7.53±1.74	5.07±1.02
**Total**	**15**	**27**			**907**	**0.719±0.053**	**0.739±0.049**	**6.95±1.52**	**4.71±1.1**

Summary statistics of the genetic diversity of 27 Creole breeds; geographical breed group, country of origin, breed names and acronyms (acron), FAO risk status, sample sizes (N), observed (H_o_) and unbiased expected (H_e_) heterozygosities, mean number of alleles (MNA), and allelic richness corrected for sample size (R_t_) are shown; SD = standard deviation; *n =* not applicable.

**Table 2:** Within-group genetic diversity of the geographical breed groups

**Geographical breed groups**	**Nb of breeds**	**N**	**H**_**o**_**±SD**	**H**_**e**_**±SD**	**MNA±SD**	**R**_**t**_**±SD**
Creole1	4	175	0.679±0.078	0.748±0.070	8.84±2.63	7.34±1.87
Creole2	4	121	0.740±0.078	0.777±0.071	9.89±2.26	8.61±1.93
Creole3	2	50	0.671±0.123	0.708±0.116	6.42±1.46	6.34±1.45
Creole4	2	61	0.692±0.120	0.745±0.096	8.37±1.67	7.88±1.52
Creole5	6	212	0.712±0.091	0.777±0.079	10.84±2.34	8.76±1.96
Creole6	9	288	0.756±0.080	0.819±0.064	12.47±3.39	10.03±2.46
Iberian1	12	609	0.674±0.106	0.748±0.110	10.79±3.54	8.09±2.15
Iberian2	10	452	0.697±0.081	0.756±0.081	11.16±2.75	8.32±1.96
Iberian3	2	100	0.681±0.124	0.732±0.093	8.42±2.29	7.40±1.82
Iberian4	16	763	0.670±0.086	0.769±0.086	11.63±3.29	8.70±2.11
British	5	200	0.653±0.079	0.754±0.067	8.89±2.21	7.21±1.76
Continental Europe	4	184	0.720±0.078	0.760±0.085	9.89±3.45	8.23±2.52
Indicine	6	168	0.654±0.111	0.735±0.112	11.32±3.16	9.14±2.49
**Total**	**82**	**3383**	**0.692±0.032**	**0.756±0.027**	**9.92±1.67**	**8.28±2.22**

Summary statistics of the genetic diversity of 13 geographical breed groups; group names, number of breeds (Nb of breeds), sample sizes (N), observed (H_o_) and unbiased expected (H_e_) heterozygosities, mean number alleles (MNA), and allelic richness corrected for sample size (R_t_) are shown; SD = standard deviation.

**Figure 1:**
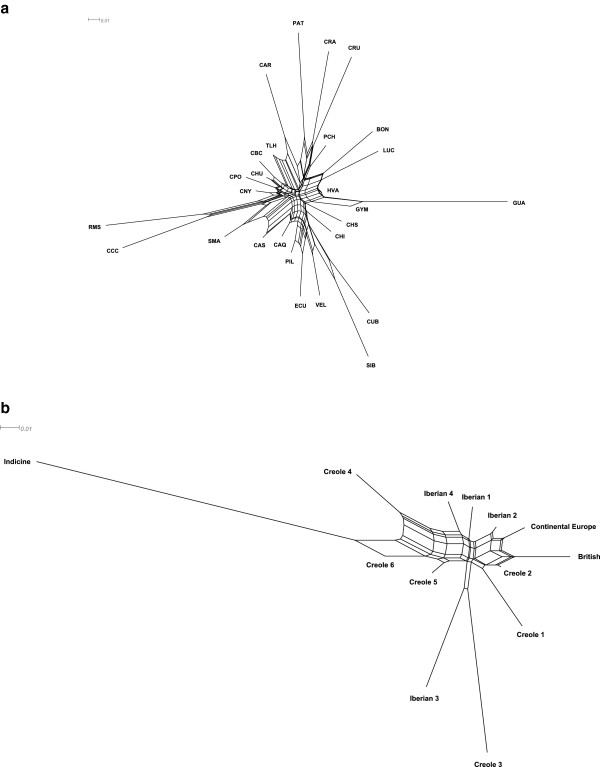
**Neighbor-net graph of kinship genetic distances.** The genetic relationships among 27 Creole cattle breeds **(a)** and 13 geographical breed groups **(b)** are shown; breed acronyms are defined as follows: CRA, Cr.Argentino; PAT, Cr. Patagonico; CAR, Caracú; CRU, Cr. Uruguayo; BON, Blanco Orejinegro; HVA, Hartón del Valle; LUC, Lucerna; PCH, Pampa Chaqueño; CCC, Costeño con Cuernos; RMS, Romosinuano; GUA, Guabalá; GUY, Guaymí; SMA, Sanmartinero; CBC, Cr. Baja California; CHU, Cr. Chihuahua; CNY, Cr. Nayarit; CPO, Cr. Poblano; TLH, Texas Longhorn; CAQ, Caqueteño; CHS, Chino Santandereano; CAS, Cr. Casanareño; VEL, Velasquez; CUB, Cr. Cubano; SIB, Siboney; ECU, Cr. Ecuatoriano; CHI, Cr. Chiapas; PIL, Cr. Pilcomayo.

The neighbor-net phylogeny of kinship distances (Figure 1a) shows the relationships among the Creole breeds. Interestingly, this phylogeny was similar to that derived from more traditional genetic distances (e.g. Reynolds distances [44]). Creole cattle from the southern regions of South America (Cr. Patagonico, Cr. Argentino and Cr. Uruguayo) were clustered together with long branch lengths, like the Colombian breeds Romosinuano and Costeño con Cuernos on one side, and Guabalá and Guaymí from Panama on the other. These breeds all share ancestral signatures of Iberian and African cattle [40]. The Caracú breed from Brazil and the Texas Longhorn breed from the United States were also clustered together in agreement with their common Iberian ancestry. Clearly, the Creole breeds Siboney, Cr. Cubano, Velasquez, Cr. Ecuatoriano, Cr. Pilcomayo, Caqueteño, Cr. Chiapas, Chino Santandereano, Cr. Casanareño, and Sanmartinero that clustered together share some common ancestry with zebu; they are known to have been crossed with indicine cattle. The majority of the Creole breeds from Mexico clustered together, whereas three breeds from Colombia (Blanco Orejinegro, Lucerna and Hartón del Valle) that have been crossed with commercial British cattle formed another cluster.

The neighbor-net graph of geographical breed clusters in Figure 1b that was derived from kinship distances illustrates these distinct admixture patterns found in Creole cattle. For example, Creole3, which includes the Costeño con Cuernos and Romosinuano breeds (from Colombia), clustered with cattle from the Canary islands of Iberian ancestry, whereas Creole2 was closely associated with commercial breeds of British origin.

**Figure 2:**
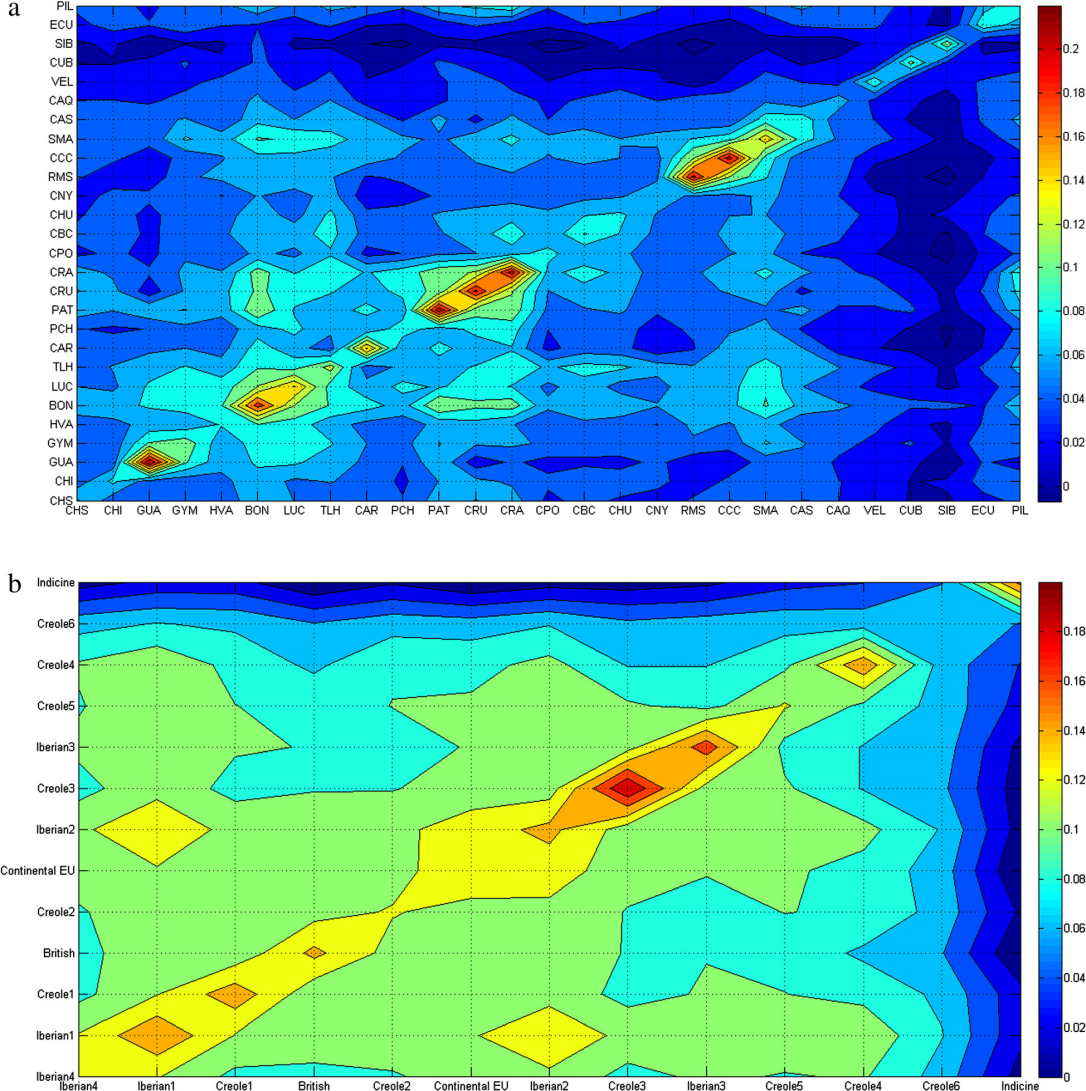
**Contour plots of marker-estimated kinships (MEK).** Creole cattle breeds **(a)** and geographical breed groups **(b)** were classified according to their respective kinship phylogenies; breed acronyms are defined as follows: CRA, Cr.Argentino; PAT, Cr. Patagonico; CAR, Caracú; CRU, Cr. Uruguayo; BON, Blanco Orejinegro; HVA, Hartón del Valle; LUC, Lucerna; PCH, Pampa Chaqueño; CCC, Costeño con Cuernos; RMS, Romosinuano; GUA, Guabalá; GUY, Guaymí; SMA, Sanmartinero; CBC, Cr. Baja California; CHU, Cr. Chihuahua; CNY, Cr. Nayarit; CPO, Cr. Poblano; TLH, Texas Longhorn; CAQ, Caqueteño; CHS, Chino Santandereano; CAS, Cr. Casanareño; VEL, Velasquez; CUB, Cr. Cubano; SIB, Siboney; ECU, Cr. Ecuatoriano; CHI, Cr. Chiapas; PIL, Cr. Pilcomayo.

The levels of within-breed diversity can also be assessed using kinship coefficients with either the MEK obtained from individual genotypes or average coancestries (*f*_m_) estimated from allele frequencies. Because of the poor quality of the extracted DNA, possibly due to inappropriate hair collection (e.g. insufficient hair roots and/or inadequate preservation), no results were obtained for *BM2113*, *ETH003*, *ETH185* and *ETH225* loci in the 35 Cr. Casanareño specimens. Therefore, this breed was excluded from the calculation of allelic richness and from average coancestry analyses because absence of genotyping data for one breed prevents the MOLKIN software from generating complete results for the other breeds (namely between-breed coancestries of Cr. Casanareño and the other Creole populations). In order to visualize both within- and between-breed kinships, contour plots were drawn in which populations were sorted according to their genetic proximity defined in the phylogenetic neighbor-net graph (Figure 2a and [see Additional file 2: Figure S1a]). Red areas represent highly inbred Creole breeds i.e. Guabalá (MEK = 0.221 and *f*_m_ = 0.354); Blanco Orejinegro (MEK = 0.191 and *f*_m_ = 0.319); Cr. Patagonico (MEK = 0.215 and *f*_m_ = 0.339); Cr. Uruguayo (MEK = 0.205 and *f*_m_ = 0.335); Cr. Argentino (MEK = 0.209 and *f*_m_ = 0.330); Romosinuano (MEK = 0.206 and *f*_m_ = 0.344); and Costeño con Cuernos (MEK = 0.210 and *f*_m_ = 0.344), while yellow areas represent breeds with intermediate kinship values i.e. Caracú (MEK = 0.163 and *f*m = 0.300), Lucerna (MEK = 0.162 and *f*m = 0.302) and Sanmartinero (MEK = 0.152 and *f*m = 0.300).

These contour plots also reveal breed relationships with related breeds clustered in different yellow and green areas, i.e. Guabalá and Guaymí; Blanco Orejinegro, Lucerna and Texas Longhorn; Blanco Orejinegro, Cr. Patagonico, Cr. Uruguayo and Cr. Argentino; Blanco Orejinegro and Sanmartinero; and Romosinuano, Costeño con Cuernos and Sanmartinero. Finally, zebu-influenced breeds in dark blue areas (Caqueteño, Velasquez, Cr. Cubano, Siboney, Cr. Ecuatoriano and Cr. Pilcomayo) are clearly separated from all others.

The geographical breed clusters with the highest within-group kinship values (red areas in Figure 2b and [see Additional file 2: Figure S1b]) were Creole3 (MEK = 0.200 and *f*_m_ = 0.297), which includes the threatened Colombian breeds Costeño con Cuernos and Romosinuano, and Iberian3 (MEK = 0.172 and *f*_m_ = 0.271), which includes two isolated breeds from the Canary Islands. Interestingly, the group of Indicine breeds, mainly composed of widely spread commercial zebu cattle, also had high kinship coefficients (MEK = 0.167 and *f*_m_ = 0.272), which suggests the existence of a substructure within this group.

Fairly high kinship coefficients were observed between Iberian1 (mostly Portuguese breeds) and Iberian2 (mostly Spanish breeds), between the Continental European group and Iberian2, and between Iberian3 (Canary Islands) and Creole3 (Colombia), as shown by the yellow areas in Figure 2b. Overall, the separation was greatest between the group of Zebu-influenced breeds in dark blue areas and the remaining groups followed by the separation between Creole6 and Creole4 with a higher degree of zebu admixture in light blue areas.

### Analyses of conservation priorities of Creole cattle

The results of the analyses of conservation priorities of Creole breeds are in Tables 3 and 4. The kinship-based methods, namely MEKs, *f*_m_ and WLM, resulted in a considerable number of Creole breeds with a null contribution to the overall genetic diversity (15, 14 and 17 breeds, respectively, out of 27). Consequently, only highly prioritized breeds can be easily identified i.e. Siboney, Cr. Cubano, Pampa Chaqueño and Cr. Poblano (0.109 < MEKs < 0.215, 0.100 <*f*_m_ < 0.251 and 0.052 < WLM <0.214) and the results were similar with the three methods applied. These may reflect the high within-breed genetic diversity of these breeds, possibly due to past crossbreeding. The WLMM method, which allows only one null contribution (in this case the Creole Blanco Orejinegro), separated breeds more effectively and prioritized for conservation the Casanareño, Siboney, Cr. Cubano, Velasquez and Cr. Ecuatoriano breeds (0.56 < WLMM < 0.107). Nevertheless, the contrasting results for the Casanareño breed (MEK = 0, no data for *f*_m_ and high WLM and WLMM) must be interpreted with caution, because of the lack of results for four loci in this breed, as explained above. The breeds Pampa Chaqueño, Cr. Nayarit, Caracú, and Cr. Chiapas had intermediate conservation values (0.042 < WLMM < 0.048).

**Table 3:** Analyses of conservation priorities of Creole cattle breeds

**Group**	**Breed**	**MEKs**	***f***_**m**_	**WLM**	**WLMM**	**PC**_**He**_	**PC**_**Weitz**_	^**1**^**PC**_**Fst**_	**PC**_**5:1**_
Creole1	CRA	0	0	0	0.012	-0.320	4.97	0.141	4.087
Creole1	PAT	0	0	0	0.011	-0.359	**5.95**	0.190	**4.896**
Creole1	CAR	0.046	0.067	0	0.042	-0.145	**5.39**	0.337	**4.466**
Creole1	CRU	0	0	0	0.014	-0.338	5.28	0.151	4.342
Creole2	BON	0	0	0	0	-0.222	3.67	0.116	3.020
Creole2	HVA	0	0	0	0.028	**0.226**	1.19	0.310	1.029
Creole2	LUC	0	0	0	0.026	-0.117	3.40	0.189	2.813
Creole2	PCH	**0.109**	**0.105**	**0.116**	0.048	0.165	2.35	0.355	1.985
Creole3	CCC	0	0	0	0.019	-0.357	5.38	0.143	4.422
Creole3	RMS	0.033	0.054	0.009	0.034	-0.366	**6.18**	0.203	**5.087**
Creole4	GUA	0.011	0.039	0.003	0.034	-0.411	**9.88**	**0.484**	**8.161**
Creole4	GYM	0	0	0	0.025	0.085	2.67	0.310	2.238
Creole5	SMA	0	0	0	0.011	-0.093	3.38	0.209	2.800
Creole5	CBC	0	0	0	0.028	0.110	1.98	0.272	1.668
Creole5	CHU	0	0	0.005	0.037	**0.196**	1.09	0.273	0.941
Creole5	CNY	0.063	0.066	0	0.045	**0.252**	1.78	**0.385**	1.525
Creole5	CPO	**0.167**	**0.100**	**0.052**	0.038	0.180	1.52	0.297	1.296
Creole5	TLH	0	0	0	0.015	0.002	1.70	0.150	1.416
Creole6	CAQ	0.046	0	0	0.034	**0.249**	1.29	0.340	1.116
Creole6	CAS	0	*n.*	**0.431**	**0.107**	0.138	1.88	0.290	1.589
Creole6	CHS	0.028	0.016	0	0.035	0.192	2.32	0.378	1.965
Creole6	VEL	0.063	0.069	0.016	**0.056**	0.157	3.08	**0.411**	2.592
Creole6	CUB	**0.124**	**0.114**	**0.123**	**0.076**	0.113	3.96	**0.448**	3.318
Creole6	SIB	**0.215**	**0.251**	**0.214**	**0.092**	0.120	**5.40**	**0.579**	**4.518**
Creole6	ECU	**0.096**	**0.084**	0.032	**0.056**	0.168	2.51	0.372	2.119
Creole6	CHI	0	0.033	0	0.042	**0.222**	1.91	0.369	1.628
Creole6	PIL	0	0	0	0.037	0.152	1.69	0.286	1.433

Contributions of 27 breeds to overall genetic diversity of Creole cattle according to: marker-estimated kinships (MEK), average coancestries (*f*_m_), weighted log-linear model (WLM), weighted log-linear mixed model (WLMM), Weitzman formula (PC_Weitz_), proportional variation of expected heterozygosity (PC_He_), aggregate diversity (PC_Fst_), and the Piyasatian and Kinghorn formula (PC_5:1_); values for the five breeds with the highest contributions are shown in bold; geographical breed groups and breed acronyms are defined in Table 1; ^1^aggregate diversity was calculated as: PC_Fst_ = PC_Weitz_*0.087+PC_He_*0.913; *n =* not applicable.

**Table 4:** **Contributions of Creole cattle breeds to diversity according to Caballero and Toro**[20]**and Fabuel et al.**[35]

**Group**	**Breed**	***f***_**ii**_	**D**_**Nei**_	^**1**^**Contribution to *f***	^**1**^**Contribution to D**	**GDT|i**	^**1**^**loss/gain (%)**	^**1**^**PC**_**weighted**_	^**2**^**PC**_**unweighted**_
Creole1	CRA	**0.329**	0.094	**0.014**	**0.044**	0.806	0.2	**5.430**	3.643
Creole1	PAT	**0.339**	**0.106**	0.009	0.031	0.805	0.1	3.813	3.655
Creole1	CAR	0.300	0.097	**0.011**	**0.043**	0.803	-0.1	5.321	3.798
Creole1	CRU	**0.335**	0.097	**0.012**	0.038	0.805	0.1	4.656	3.632
Creole2	BON	0.319	0.084	0.007	0.022	0.805	0.1	2.716	3.644
Creole2	HVA	0.234	0.058	0.004	0.021	0.804	0.0	2.578	3.931
Creole2	LUC	0.302	0.081	0.006	0.021	0.804	0.0	2.656	3.713
Creole2	PCH	0.237	0.065	**0.010**	**0.048**	0.803	-0.1	**5.884**	3.948
Creole3	CCC	**0.344**	**0.111**	0.007	0.022	0.804	0.0	2.727	3.659
Creole3	RMS	**0.344**	**0.118**	0.006	0.022	0.804	0.0	2.750	3.690
Creole4	GUA	**0.354**	**0.127**	0.007	0.022	0.803	-0.1	2.745	3.683
Creole4	GYM	0.255	0.066	0.008	0.034	0.804	0.0	4.151	3.868
Creole5	SMA	0.298	0.080	0.006	0.022	0.804	0.0	2.778	3.728
Creole5	CBC	0.266	0.071	0.005	0.019	0.804	0.0	2.401	3.835
Creole5	CHU	0.246	0.067	0.004	0.018	0.804	0.0	2.216	3.913
Creole5	CNY	0.229	0.066	0.004	0.023	0.803	-0.1	2.852	**3.987**
Creole5	CPO	0.236	0.063	0.008	**0.041**	0.803	-0.1	5.057	3.945
Creole5	TLH	0.266	0.064	**0.019**	**0.073**	0.806	0.2	**9.060**	3.799
Creole6	CAQ	0.233	0.064	0.005	0.024	0.803	-0.1	2.952	3.961
Creole6	CHS	0.236	0.068	0.005	0.024	0.803	-0.1	2.956	3.967
Creole6	VEL	0.249	0.085	0.005	0.024	0.803	-0.1	2.966	**3.981**
Creole6	CUB	0.251	0.095	0.009	**0.048**	**0.801**	**-0.4**	**5.999**	**4.025**
Creole6	SIB	0.246	**0.108**	0.008	**0.049**	**0.799**	**-0.6**	**6.123**	**4.108**
Creole6	ECU	0.262	0.092	0.002	0.011	0.803	-0.1	1.414	3.954
Creole6	CHI	0.226	0.066	0.006	0.029	0.803	-0.1	3.578	**4.001**
Creole6	PIL	0.242	0.068	0.007	0.034	0.804	0.0	4.222	3.935

Average coancestries (*f*_ii_) and Nei’s genetic distances (D_Nei_), contributions to global coancestry (*f*) and to average Nei’s distance (D), global coancestry (GDT|i) and proportional loss or gain in genetic diversity after removing each breed, proportional contributions (PC) to a pool of maximum genetic diversity weighted and unweighted by sample sizes; values for the five breeds with the highest contributions are shown in bold, except for GDT|i and loss/gain for which only the two highest values are in bold.; geographical breed groups and breed acronyms are defined in Table 1; ^1^average coancestries weighted by sample sizes; ^2^average coancestries estimated by ignoring sample sizes; mean coancestry within-breeds, *f* = 0.275; mean Nei’s minimum distance in the metapopulation, D = 0.083; mean coancestry in the metapopulation, *f* = 0.197; global genetic diversity of the metapopulation, GDT = 0.804.

The proportional contribution of each breed to the average heterozygosity of the metapopulation, resulted in many negative values (10 breeds), which, if these breeds were removed, would lead to a 'gain’ in diversity. In accordance with their inbred status (high within-breed kinship coefficients), the breeds Guabalá, Romosinuano, Cr. Patagonico and Costeño con Cuernos had the most negative PC_He_ values (between -0.411 and -0.357). This method ranked breeds with greater H_e_ values at a higher level, such as Cr. Nayarit, Caqueteño, Hartón del Valle, Cr. Chiapas and Cr. Chihuahua (0.196 < PC_He_ < 0.252). The breeds Chino Santandereano, Cr. Poblano, Cr. Ecuatoriano, Pampa Chaqueño, Velasquez and Cr. Pilcomayo had intermediate contributions (0.152 < PC_He_ < 0.192). Although Cr. Casanareño was excluded from the coancestry analysis, it resulted in a null MEK contribution and was highly prioritized by the WLM and WLMM as a consequence of having a significant effect (~14%) in the overall heterozygosity of Creole cattle.

The Weitzman approach prioritizes highly differentiated breeds (i.e. those with large genetic distances from all others) based only on their contribution to between-breed genetic diversity. In this case, breeds with the highest contributions (5.39 < PC_Weitz_ < 9.88) were Guabalá, Romosinuano, Cr. Patagonico, Siboney and Caracú followed by Costeño con Cuernos, Cr. Uruguayo and Cr. Argentino (5.38 < PC_Weitz_ < 4.97). Mexican breeds had the lowest contributions amongst all breeds (< 2%).

The combined approach of Ollivier and Foulley [37] (PC_Fst_), which takes into account both within- and between-breed components of the genetic diversity, seems to provide more balanced solutions. In this case, the between-breed component (i.e. PC_Weitz_) was weighted by the overall F_st_ value of 0.087 obtained for the metapopulation of Creole breeds. The PC_Fst_ approach prioritized Creole breeds that also ranked high with the kinship-based methods (i.e. with high within-breed diversity), namely Siboney, Cr. Cubano, Velasquez and Cr. Nayarit (0.385 < PC_Fst_ < 0.579). Nevertheless, some of the breeds prioritized by PC_Weitz_ (i.e*.* with greater genetic distances) also had high PC_Fst_ estimates, particularly Guabalá, Caracú and the above mentioned Siboney (0.337 < PC_Fst_ < 0.579). Creole breeds Chino Santandereano, Cr. Ecuatoriano, Cr. Chiapas, Caqueteño, Pampa Chaqueño, Hartón del Valle and Guaymí had intermediate conservation values (0.310 < PC_Fst_ < 0.378). The PC_5:1_ method gave the same results in terms of breed ranking for conservation priorities as the Weitzman approach.

The results of the combined approach of Caballero and Toro [20] and Fabuel et al. [35] are in Table 4. The Texas Longhorn breed had the greatest contribution to global coancestry (*f*, 0.019) because its within-breed coancestry was quite high (*f*_ii_ = 0.266) and its distance from all the other populations was among the lowest observed (D_Nei_ = 0.064). The breeds Cr. Argentino, Caracú and Cr. Uruguayo also had high contributions to *f* (0.014, 0.012 and 0.011, respectively) as a consequence of their high *f*_ii_ values (between 0.300 and 0.329). Although the Cr. Patagonico, Guabalá, Costeño con Cuernos and Romosinuano breeds had high *f*_ii_ values (between 0.339 and 0.354), their mean genetic distances were also rather large (between 0.106 and 0.127) and thus their contributions to *f,* obtained from the difference between *f*_ii_ and D_Nei_*,* were less significant (0.009, 0.007, 0.007 and 0.006, respectively). The Texas Longhorn breed had the highest contribution to D (0.073) and to proportional contribution estimates (PC_weighted_, 9.060), but these estimates can be biased as a consequence of the rather large sample size of this population (N = 80), which is taken into account in all calculations. This could also be valid for the PC estimates of the breeds Siboney (6.123), Cr. Cubano (5.999), Pampa Chaqueño (5.884) e Cr. Argentino (5.430), which also had large samples sizes. Indeed, when the proportional contributions to genetic diversity are estimated by ignoring sample sizes (PC_unweighted_), only the Siboney and Cr. Cubano breeds maintain their high ranking (4.108 and 4.025, respectively), followed by Cr. Chiapas (4.001), Cr. Nayarit (3.987) and Velasquez (3.981). The Caracú, Cr. Poblano, Cr. Uruguayo, Cr. Pilcomayo, Guaymí and Cr. Patagonico breeds had quite high proportional contributions to genetic diversity (3.813 < PC_weighted_ <5.321), whereas Cr. Ecuatoriano had the lowest contribution (PC_weighted_ = 1.414), perhaps because of its low representation (N = 12). The proportional contribution of each breed to a pool of maximum genetic diversity showed very little variation among Creole breeds, but removal of the Siboney and Cr. Cubano breeds from the metapopulation of Creole cattle caused the greatest loss in total genetic diversity (-0.6% and -0.4%, respectively).

### Analyses of conservation priorities of breed groups

The results of the analyses of conservation priorities of geographical breed-groups are in Tables 5 and 6. With the kinship-based methods (MEKs, *f*_m_, WLM and WLMM), the breed groups Creole6 (zebu-influenced), Indicine, British and Iberian4 (most Spanish breeds) were highly prioritized (0.064 < MEKs < 0.427, 0.056 <*f*_m_ < 0.402, 0.049 < WLM <0.546, 0.087 < WLMM < 0.257) because of the rather high within-group genetic diversity which reflects their heterogeneous genetic make-up. Based on the MEKs and *f*_m_ estimates, Creole3 (Costeño con Cuernos and Romosinuano breeds from Colombia) had an intermediate conservation value (0.053 and 0.066, respectively). Similarly, with WLM and WLMM, the Iberian3 (breeds from the Canary Islands) and Continental European (WLM = 0.033 and WLMM = 0.076, respectively) breed groups had an intermediate position. As expected, PC_He_ estimates resulted in negative contributions for eight of the 13 geographical breed groups and prioritized Creole6, Creole2, Creole5, Iberian4 and Continental European cattle (0.041 < PC_He_ < 0.694) for conservation.

**Table 5:** Analyses of conservation priorities of geographical breed groups

**Group**	**MEKs**	***f***_**m**_	**WLM**	**WLMM**	**PC**_**He**_	**PC**_**Weitz**_	^**1**^**PC**_**Fst**_	**PC**_**5:1**_
Creole1	0	0	0	0.028	-0.084	**6.25**	0.220	**5.192**
Creole2	0	0	0	0.068	**0.228**	3.23	**0.372**	2.729
Creole3	**0.053**	**0.066**	0.025	0.052	-0.526	**16.53**	0.293	**13.682**
Creole4	0.004	0.025	0.018	0.047	-0.121	**14.22**	**0.567**	**11.825**
Creole5	0	0	0	0.044	**0.236**	3.76	**0.405**	3.172
Creole6	**0.427**	**0.402**	**0.546**	**0.146**	**0.694**	4.43	**0.873**	3.806
Iberian1	0	0	0	0	-0.090	3.87	0.100	3.209
Iberian2	0	0	0	0.021	-0.002	2.06	0.097	1.716
Iberian3	0.004	0.011	**0.033**	0.071	-0.264	**9.90**	0.224	**8.203**
Iberian4	**0.064**	**0.056**	**0.049**	**0.087**	**0.138**	3.61	0.305	3.030
British	**0.185**	**0.180**	**0.148**	**0.102**	-0.019	5.23	0.233	4.353
Continental Europe	0.019	0.020	0.010	**0.076**	**0.041**	3.54	0.209	2.956
Indicine	**0.243**	**0.241**	**0.171**	**0.257**	-0.231	**27.16**	**1.083**	**22.586**

Contributions of 13 geographical breed groups to the overall genetic diversity of worldwide cattle according to: marker estimated kinships (MEK), average coancestries (*f*_m_), weighted log-linear model (WLM), weighted log-linear mixed model (WLMM), Weitzman formula (PC_Weitz_), proportional variation of the expected heterozygosity (PC_He_), aggregate diversity (PC_Fst_), and the Piyasatian and Kinghorn formula (PC_5:1_); values for the five breed groups with the highest contributions are shown in bold; breeds in each group are defined in Table 1;^1^aggregate diversity was calculated as: PC_Fst_ = PC_Weitz_*0.048+PC_He_*0.952.

**Table 6:** **Contributions of geographical breed groups to diversity according to Caballero and Toro**[20]**and Fabuel et al.**[35]

**Group**	***f***_**ii**_	**D**_**Nei**_	^**1**^**Contrib to *f***	^**1**^**Contrib to D**	**GDT|i**	^**1**^**loss/gain (%)**	^**1**^**PC**_**weighted**_	^**2**^**PC**_**unweighted**_
Creole1	**0.254**	0.051	0.010	0.041	0.795	0.0	5.109	7.597
Creole2	0.228	0.038	0.007	0.029	0.795	0.0	3.587	**7.715**
Creole3	**0.297**	**0.075**	0.003	0.012	0.795	0.0	1.425	7.418
Creole4	**0.262**	**0.061**	0.004	0.014	0.795	0.0	1.786	7.619
Creole5	0.228	0.039	**0.012**	**0.051**	0.795	0.0	**6.300**	**7.733**
Creole6	0.183	0.041	**0.012**	**0.073**	**0.790**	**-0.6**	**9.046**	**8.174**
Iberian1	0.253	0.041	**0.038**	**0.142**	0.801	0.8	**17.569**	7.507
Iberian2	0.246	0.039	**0.028**	**0.106**	0.798	0.4	**13.117**	7.552
Iberian3	**0.271**	**0.061**	0.006	0.023	0.795	0.0	2.895	7.533
Iberian4	0.233	0.043	**0.043**	**0.183**	0.796	0.1	**22.623**	**7.716**
British	0.247	**0.051**	**0.012**	0.048	0.794	-0.1	5.892	**7.667**
Continental Europe	0.243	0.044	**0.011**	0.044	0.795	0.0	5.392	7.626
Indicine	**0.272**	**0.127**	0.007	0.042	**0.787**	**-1.0**	5.258	**8.144**

Average coancestries (*f*_ii_) and Nei’s genetic distances (D_Nei_), contributions to global coancestry (*f*) and to average Nei’s distance (D), global coancestry (GDT|i) and proportional loss or gain in genetic diversity after removing each breed group, proportional contributions (PC) to a pool of maximum genetic diversity weighted and unweighted by sample sizes are shown; values for the five breed groups with the highest contributions are shown in bold, except for GDT|i and loss/gain for which only the two highest values; breeds in each group are defined in Table 1; ^1^average coancestries weighted by sample sizes; ^2^average coancestries estimated by ignoring sample sizes; mean coancestry within-groups, *f* = 0.241; mean Nei’s minimum distance in the metapopulation, D = 0.055; mean coancestry in the metapopulation, *f* = 0.205; global genetic diversity of the metapopulation, GDT = 0.795.

Indicine cattle had a particularly high Weitzman estimate (PC_Weitz_ = 27.16) which reflected their wide phylogenetic differentiation. This approach also prioritized Creole3 (16.53) and Creole4 (14.22), whereas Iberian3 and Creole1 had more intermediate values (9.90 and 6.25, respectively). The strong differentiation of these geographical breed groups is confirmed by their large genetic distances, as shown [see Additional file 3: Figure S2].

Given the inherent limitations of the Weitzman procedure, especially when the number of breeds is large, the alternative approximation algorithm of Garcia et al. [22] was also tested with the 13 geographical breed groups [see Additional file 4: Figure S3] to investigate the partial contributions of each breed to genetic diversity. Our results indicate that the approximate estimates of Weitzman and exact methods are in full agreement only for thresholds between 0.300 and 0.200. Furthermore, these results suggest that it would not be practical to carry this type of analysis of conservation priorities of worldwide cattle at the breed level (too many breeds, too computationally demanding), even if the approximate method is used. These results provide additional support to our approach that considers geographical breed groups instead of individual breeds.

The strategy based on the analysis of breed groups produced an overall F_st_ value of 0.048, which was used in the formula of Ollivier and Foulley [37] (PC_Fst_ = PC_Weitz_*0.048 + PC_He_*0.952) to calculate conservation values. With this method, well-differentiated breed groups such as Indicine and Creole6 were prioritized (1.083 and 0.873, respectively), but Creole4, Creole5 and Creole2 also ranked high (0.372 < PC_Fst_ < 0.567). According to this method, Iberian2 (a group of Iberian breeds with a recognized admixed background) had the lowest conservation value (0.097). Both Piyasatian and Kinghorn [34] and Weitzman approaches gave the same results in terms of ranking of conservation priorities, as shown in the analysis of individual Creole breeds.

The results of the combined approach of Caballero and Toro [20] and Fabuel et al. [35] for the geographical breed groups are in Table 6. Contributions to global coancestry were high for Iberian4, Iberian1 and Iberian2 (between 0.028 and 0.038), whereas they were moderate for the British, Creole5, Creole6 and Continental European groups (about 0.012). Their contributions to D were also high (between 0.051 and 0.183) due to their lower average Nei’s minimum distances. Accordingly, the above-mentioned Iberian breed groups were highly prioritized when samples sizes were considered (13.117 < PC_weighted_ < 22.623). Indicine and Creole6 had the most negative impact on overall genetic diversity when they were removed from the metapopulation (GDT|I = 0.787, loss/gain = -1.0% and GDT|I = 0.790, loss/gain = -0.6%, respectively). When the proportional contributions to genetic diversity were estimated ignoring sample sizes (PC_unweighted_), Indicine, Creole6, Creole5, Iberian4, Creole2 and British breed groups ranked highest (7.715 < PC_unweighted_ < 8.174).

## Discussion

The need for conservation of genetic resources for food and agriculture is widely recognized [1], but the existence of organized programs for the conservation of domestic animal breeds differs considerably between countries. With a few exceptions, e.g. [53-56], systematic conservation actions have been taken for Creole cattle breeds (but not all) only in the last few years [1]. While several Creole breeds are considered 'not at risk’ according to the FAO risk categories (Table 1), no information on the number of breeding animals and on their population trend is available for most of these breeds. Creole populations are descendants of Iberian cattle brought to the Americas nearly five centuries ago [57]; the process of separation and divergence of these populations with many generations of genetic drift has probably determined their retention of genetic diversity and adaptation to a very wide range of environmental conditions [40]. However, many of these Creole breeds have been abandoned or extensively crossed with commercial European and zebu breeds and conservation programs are necessary to avoid further losses of genetic diversity.

In many cases, a conservation program will concern many breeds while financial resources are limited, thus the need to set priorities. Factors that need to be considered when defining such priorities include the importance of a breed in terms of genetic uniqueness but also its own genetic diversity, and other aspects such as adaptation to specific environments, possession of unique traits, cultural and historical value, contribution to environmental sustainability, etc. [58]. Once priorities have been established, different conservation strategies can be applied, namely *in situ* or *ex situ in vivo* preservation, and cryoconservation, which differ in their ability to address the different aspects considered in the rationale for conservation [59].

Although other factors that can potentially impact conservation decisions are important, knowledge of the population structure of a livestock species in terms of distribution of genetic variability within- and between-breeds is a key factor to establish conservation priorities and strategies [20], that aim at maintaining genetic diversity for future generations [60]. Previous studies [40,43,44,61] have confirmed that Creole breeds retain high levels of genetic diversity, and that they differ considerably from each other and from their Iberian ancestors. Some of this diversity may result from admixture with exotic germplasm in a number of Creole breeds, such as those from Cuba and Ecuador, which have been influenced by admixture from zebu cattle over the last century [40].

Our results indicate that under different conservation perspectives, the groups of Creole breeds generally had a higher conservation priority than Iberian, British or Continental European breed groups (Tables 5 and 6). This may reflect the strong selection pressure and consequent loss of genetic diversity of commercial European breeds over the years, such as those included in the British and Continental European breed groups, whereas herdbooks and organized breeding programs have only recently been adopted for a few Creole breeds. Among the Creole breeds, conservation priorities depended on whether the method used placed more emphasis on the contribution of each breed to the within- or the between-breed component of genetic diversity. Thus, if the focus was on breed distinctiveness, priority was given to most breeds classified in breed groups Creole1 (breeds from Argentina, Brazil and Uruguay), Creole3 (two breeds from Colombia) and Creole4 (breeds from Panama), whereas if the focus was on within-breed diversity, priority was given to breeds from Creole2 (three breeds from Colombia and one from Paraguay), Creole5 (most Mexican breeds) and Creole6 (two breeds from Cuba, one from Ecuador, one from Mexico, and most Colombian breeds). Finally, for individual Creole breeds, the contribution to genetic diversity based on average coancestries combined with genetic distances (Table 4), generally prioritized breeds that are thought to have the highest degree of zebu admixture (Creole5 and Creole6).

The difficulties associated with the choice of the best method to prioritize breeds for conservation decisions were recently discussed in several papers [11,24,27,39] and are illustrated by our results. For example, Martinez et al. [40] reported that the highly-threatened Guabalá is a Creole breed with strong signatures of Iberian ancestry. However, in our study, it had the lowest levels of genetic diversity, the highest within-breed coancestry, and the highest average Nei’s distance (Tables 1 and 4). Thus, including the Guabalá breed among those prioritized for conservation depended highly on the criteria used for breed ranking, i.e. it was classified as a top priority when the focus was on between-breed diversity (such as in the Weitzman procedure), but was the first breed to be excluded when only the within-breed component of genetic diversity was considered (proportional contribution to H_e_). This is in line with previous findings [16,17] that indicate that small, inbred breeds will be given priority when the emphasis is placed on the between-breed component. This result invalidates the use of the Weitzman approach, based on genetic distances, as a single measure for breed prioritization. In contrast, higher ranking will be given to large, and possibly crossbred, populations when the emphasis is placed on the within-breed component [24]. Nevertheless, when other methods are used, the ranking of Guabalá depends on the weight given to each component of the overall genetic diversity, i.e. it has a low conservation value with kinship-based methods (Table 3) because of its high inbreeding but it is highly ranked with methods that give greater weight to the between-breed component such as the Piyasatian and Kinghorn approach [34]. When analyzing the genetic diversity of Iberian cattle, Cañon et al. [39] were faced with the same difficulty, i.e. that the more a breed was differentiated (e.g. Mirandesa) the more likely it had undergone strong genetic drift and showed high levels of inbreeding. Thus, classifying such breeds among the top priorities for conservation decisions depended on the strategy followed, although they constitute reservoirs of rare alleles. This pattern of genetic variation distribution is typical of sub-divided populations in which the global genetic diversity of the species is maintained at the cost of a loss in the genetic variability of the sub-populations.

Overall, the choice of the most appropriate method to prioritize breeds for conservation decisions is determined by whether it is important to maintain genetic diversity for the short- or long-term. For example, if the focus is on short-term objectives, the emphasis should be placed on maintaining high levels of heterozygosity, while if it is on long-term objectives, the emphasis should be placed on allelic richness and breed differentiation [9]. In the particular case of Creole cattle, for which distinct evolutionary trajectories and selection for adaptation to extreme environments have played a major role, the preservation of high levels of allelic diversity is a key element for their long-term preservation and for maintaining their ability to cope and adjust to future environmental changes. Furthermore, in addition to establishing conservation priorities among candidate breeds, it may be important to carry out similar analyses on a within-breed basis to define conservation priorities among different herds, strains, etc., which may be particularly important in breeds with a pronounced substructure [39].

One key issue, which is well-exemplified in the case of Creole cattle, is how misleading the exclusive consideration of high heterozygosity might be, and how it should be carefully assessed and interpreted. For example, several of the Creole breeds included in our study are known to have been admixed with zebu cattle in the beginning of the 20th century, and show strong signs of this influence [40,61]. As expected, breeds with zebu admixture (which are mainly included in the Creole6 breed group) have the highest levels of heterozygosity (Table 1), and thus are often classified as the top priority for conservation decisions (Tables 3 and 4). Indeed, this is also in the case in the analyses of the geographical breed groups, which classify Creole6 and Indicine as the top priorities for conservation with most of the methods used (Table 5). These results reinforce the idea that statistical analyses aimed at making conservation decisions are useful but should be considered carefully, since there is a risk that some breeds or breed groups may be ignored in conservation programs. Thus, such decisions must take into account additional factors, including the results of other methods such as cluster and admixture analyses. One example of an ancillary method is the estimation of marginal kinship-based diversities per breed [16], which take into account extinction probabilities, to measure the expected loss of diversity within a defined time interval. Given the difficulties with estimating valid extinction probabilities [12] for most of the Creole breeds, for which the risk status is largely unknown, we chose not to include this approach in our analyses but recognize its relevance to conservation studies.

The establishment of conservation priorities based only on 'neutral’ genetic markers, such as microsatellites, can fail to take into account important genetic information associated with phenotypic variation (e.g. morphology or production traits), disease resistance, and other adaptive traits. Starting with a few *B. taurus* animals imported from Iberia since the 15^th^ century [62], Creole cattle expanded throughout the Americas and adapted to environmental conditions ranging from the Chihuahua desert to Patagonia, or from the tropical climate of the Caribbean to the Chilean Andes. Natural selection probably played a major role in the adaptation to these novel and diverse environmental niches and in the differentiation of Creole subpopulations. When compared to other taurine breeds, Creole breeds have been shown to have better heat tolerance [63,64], greater resistance to ticks [65,66] and to Tropical Ox Warble [67], lower incidence of anaplasmosis [68], better immune response [69], greater docility and more desirable grazing behavior [70], higher fertility and longevity [67], and higher productivity under given conditions [71]. This resilience can be of extreme importance in a world facing climate changes [72]. Whole-genome approaches using next-generation sequencing have been developed for livestock species, particularly cattle, which allow identification of genomic regions under selection [73-75]. Because a high number of genetic markers in coding and non-coding genomic regions can be used (e.g. SNPs), such a genomic approach can provide more reliable estimates of inbreeding coefficients when pedigree information is lacking, as well as more accurate measures of genetic diversity and of the conservation value of the different breeds [10]. Thus, and following recent FAO guidelines for the *in vivo* preservation of AnGR [76], it is important to investigate furthermore and carefully evaluate the usefulness of whole-genome SNPs to define conservation priorities of genetically distinct breeds of Creole cattle in addition to the microsatellite-based inferences reported in this study. Creole breeds have a particular evolutionary history that can be crucial to better understand the genetic basis of adaptation because, from a small number of animals originally brought from the Iberian peninsula [57], Creole cattle populations have adapted to very distinct environmental conditions, spreading from Texas to Patagonia. Thus, it can be expected that Creole cattle carry specific genetic signatures of genomic regions under selection, and genome sequencing will be extremely useful to identify these regions.

Prior to applying the conservation priority principles discussed here on a large scale, it is essential that a better and more extensive sampling is carried out, particularly for more endangered breeds, and that a general agreement is reached on the specific criteria to be used in the definition of priorities. Besides factors directly associated with genetic diversity, which have been the subject of our study, other aspects such as the contribution of a breed to food security and economic return, the demography and risk status, the existence of unique traits or specific adaptation features, the historical and cultural values, the contribution to sustainable development and environmental balance, etc., should also be taken into account [58]. The end result may be an index combining the different ranking criteria weighted appropriately to establish conservation priorities, as outlined by the FAO [59,76]. In any case, the consensus is that the best way to ensure the survival of a breed is to make its use more profitable and appealing to producers. The development of sustainable utilization and organized crossbreeding programs involving Creole breeds, and the added value resulting from their products, could make a major contribution towards their survival for the future.

## Conclusions

The contributions to within- and between-breed genetic diversity based on 'neutral’ genetic markers were evaluated in a large sample of Iberoamerican cattle breeds, to provide the basis for establishing conservation priorities. Our results indicate that Creole cattle breeds retain considerable levels of genetic diversity and that several local isolated breeds are important reservoirs of genetic diversity. Conservation priorities depended on the approach used, i.e., on whether the emphasis was placed on the within- or the between-breed component of genetic diversity. In general, if the focus was on between-breed diversity, the Creole breeds classified as top priority were the most clearly differentiated with smaller census and higher levels of inbreeding, while if the focus was on within-breed genetic diversity, they failed to be considered. Thus, besides its contribution to the overall genetic diversity, other features of a breed should also be considered when considering a conservation program, such as its adaptation to specific environments, possession of unique traits, cultural and historical value, and contribution to environmental sustainability, among others. Other types of genetic markers, including novel SNPs, may detect other genetic factors related to breed differentiation, especially those underlying adaptation and production traits, and should be investigated for use in conservation applications.

## Competing interests

The authors declare that they have no competing interests.

## Authors’ contributions

Participated in the design of the study and performed the statistical analysis: CG, LTG, OC, JVD, DG, JC. Conceived and designed the experimental assays: CG, SD, VL, IM-B, AM-M, MCTP, PZ. Carried out the assays: CG, SD, VL, IM-B, AM-M, CR. Drafted the manuscript: CG, LTG, OC, JC. Reviewed and edited the manuscript: JVD, SD, AM-M, MCTP, PZ. Members of the BIOBOVIS Consortium provided biological samples and logistic support. All authors read and approved the final manuscript.

## Authors’ information

Members of the BioBovis Consortium, http://biobovis.jimdo.com/investigadores/.

BioBovis Consortium members (listed alphabetically): Atzel Acosta, Centro Nacional de Sanidad Agropecuaria, Cuba; Luz A Álvarez, Universidad Nacional de Colombia, Colombia; Esperanza Camacho, IFAPA, Centro Alameda del Obispo, Spain; José R Marques, EMBRAPA Amazônia Oriental, Brazil; O Roberto Martínez, Centro Multidisciplinario de Investigaciones Tecnológicas, Universidad Nacional de Asunción, Paraguay; Ruben D Martínez, Facultad de Ciencias Agrarias, Universidad Nacional de Lomas de Zamora, Argentina; Guillermo Martínez-Velázquez, Instituto Nacional de Investigaciones Forestales, Agrícolas y Pecuarias, Mexico; Lilia Melucci, Universidad Nacional de Mar del Plata and Instituto Nacional de Tecnología Agropecuaria, Argentina; Jaime E Muñoz, Universidad Nacional de Colombia, Colombia; E. Armstrong, Facultad de Veterinaria, Universidad de la República, Uruguay; Jorge Quiroz, Instituto Nacional de Investigaciones Forestales, Agrícolas y Pecuarias, Mexico; Philip Sponenberg, Virginia-Maryland Regional College of Veterinary Medicine, Virginia Tech, United States of America; Odalys Uffo, Centro Nacional de Sanidad Agropecuaria, Cuba; José Luis Vega-Pla, Laboratorio de Investigación Aplicada, Cría Caballar de las Fuerzas Armadas, Spain; Axel Villalobos, Instituto de Investigación Agropecuaria, Panama; Delsito Zambrano, Universidad Técnica Estatal de Quevedo, Ecuador.

## Supplementary Material

Additional file 1: Table S1 Details on the cattle breeds and geographical breed groups included in this study. Description: Breed names axnd acronyms, sample origins and sizes, and number of genotyped microsatellite loci are provided. Summary statistics of within-breed genetic diversity are also shown, namely: observed (H_o_) and unbiased expected (H_e_) heterozygosities, mean number of alleles (MNA), allelic richness corrected for sample size (R_t_), and the respective standard deviation (SD). Click here for file

Additional file 2: Figure S1 Contour plots of average coancestries (*f*_m_). Description: Creole cattle breeds (a) and geographical breed groups (b) were sorted according to the respective kinship phylogenies. Breed acronyms are defined as follows: CRA, Cr. Argentino; PAT, Cr. Patagonico; CAR, Caracú; CRU, Cr. Uruguayo; BON, Blanco Orejinegro; HVA, Hartón del Valle; LUC, Lucerna; PCH, Pampa Chaqueño; CCC, Costeño con Cuernos; RMS, Romosinuano; GUA, Guabalá; GUY, Guaymí; SMA, Sanmartinero; CBC, Cr. Baja California; CHU, Cr. Chihuahua; CNY, Cr. Nayarit; CPO, Cr. Poblano; TLH, Texas Longhorn; CAQ, Caqueteño; CHS, Chino Santandereano; CAS, Cr. Casanareño; VEL, Velasquez; CUB, Cr. Cubano; SIB, Siboney; ECU, Cr. Ecuatoriano; CHI, Cr. Chiapas; PIL, Cr. Pilcomayo. Click here for file

Additional file 3: Figure S2 Description: Neighbor-net graph of Reynolds distances showing the genetic relationships among the 13 geographical breed groups studied. Click here for file

Additional file 4: Figure S3 Variation of breed-group contributions to Weitzman diversity using the approximation algorithm of Garcia et al. [22]. Description: This figure shows that the approximate estimates of the Weitzman diversity and exact methods are in full agreement only for thresholds between 0.300 and 0.200. Given the large number of breeds involved, applying the approximate procedure would be too computationally demanding, thus geographical breed groups were defined for the analysis of conservation priorities of worldwide cattle. Click here for file
