# Supplementary material for: Analysis of conservation priorities of Iberoamerican cattle based on autosomal microsatellite markers
Source: Genet Sel Evol. 2013 Sep 30;45(1):35. doi: 10.1186/1297-9686-45-35 (PMC3851275; doi:10.1186/1297-9686-45-35)
Supplement: Additional file 1: Table S1 — Details on the cattle breeds and geographical breed groups included in this study. Description: Breed names axnd acronyms, sample origins and sizes, and number of genotyped microsatellite loci are provided. Summary statistics of within-breed genetic diversity are also shown, namely: observed (Ho) and unbiased expected (He) heterozygosities, mean number of alleles (MNA), allelic richness corrected for sample size (Rt), and the respective standard deviation (SD). [file 1297-9686-45-35-S1.pdf]

Table S1. Details on the cattle breeds and geographical breed groups studied.

Breed names and acronyms, sample origins and sizes, and number of genotyped microsatellite loci are provided. Summary statistics of within-breed genetic diversity are also shown, namely: observed (H_o) and unbiased expected (H_e) heterozygosities, mean number of alleles (MNA), allelic richness corrected for sample size (R_t), and the respective standard deviation (SD).

Geographical breed group	Breed Name	Acronym	Sample origin	N	Loci	H_o	SD	H_e	SD	MNA	SD	R_t	SD
Creole1	Criollo Argentino	CRA	Argentina	50	19	0.673	0.110	0.678	0.101	6.26	1.66	4.00	0.80
Creole1	Criollo Patagonico	PAT	Argentina	35	19	0.629	0.124	0.670	0.108	5.32	1.57	3.84	0.84
Creole1	Caracú	CAR	Brazil	47	19	0.733	0.101	0.711	0.095	6.74	1.73	4.32	0.81
Creole1	Criollo Uruguayo	CRU	Uruguay	43	19	0.668	0.107	0.674	0.085	5.63	1.67	3.97	0.78
Creole2	Blanco Orejinegro	BON	Colombia	25	19	0.737	0.127	0.697	0.100	5.74	1.76	4.10	0.82
Creole2	Hartón del Valle	HVA	Colombia	22	19	0.783	0.130	0.783	0.070	7.74	1.73	5.24	0.92
Creole2	Lucerna	LUC	Colombia	24	19	0.673	0.152	0.717	0.108	6.63	2.06	4.69	1.11
Creole2	Pampa Chaqueño	PCH	Paraguay	50	19	0.750	0.091	0.771	0.074	8.11	1.79	5.05	0.92
Creole3	Costeño con Cuernos	CCC	Colombia	25	19	0.692	0.168	0.671	0.135	5.26	1.37	3.94	0.95
Creole3	Romosinuano	RMS	Colombia	25	19	0.651	0.140	0.669	0.132	5.11	1.59	3.94	1.00
Creole4	Guabalá	GUA	Panama	25	19	0.629	0.218	0.660	0.196	5.79	1.96	4.10	1.30
Creole4	Guaymí	GUY	Panama	36	19	0.735	0.082	0.756	0.075	7.79	1.65	4.93	0.91
Creole5	Sanmartinero	SMA	Colombia	25	19	0.692	0.124	0.721	0.079	6.37	1.16	4.39	0.73
Creole5	Criollo Baja California	CBC	Mexico	21	19	0.742	0.158	0.760	0.082	7.05	1.58	4.98	0.98
Creole5	Criollo Chihuahua	CHU	Mexico	19	19	0.719	0.168	0.777	0.080	6.68	1.49	5.14	0.95
Creole5	Criollo Nayarit	CNY	Mexico	24	19	0.749	0.121	0.788	0.077	7.74	1.94	5.25	0.89
Creole5	Criollo Poblano	CPO	Mexico	43	19	0.693	0.108	0.774	0.076	8.37	2.01	5.07	1.01
Creole5	Texas Longhorn	TLH	USA	80	19	0.707	0.117	0.740	0.111	8.05	2.46	4.78	1.13
Creole6	Caqueteño	CAQ	Colombia	25	19	0.780	0.147	0.787	0.075	7.58	1.57	5.22	0.97
Creole6	Chino Santandereano	CHS	Colombia	25	19	0.726	0.091	0.776	0.055	7.32	1.73	5.03	0.80
Creole6	Criollo Casanareño	CAS	Colombia	35	15	0.739	0.414	0.766	0.078	8.00	1.65	n.a.	n.a.
Creole6	Velasquez	VEL	Colombia	25	19	0.730	0.122	0.769	0.069	6.79	1.44	4.92	0.87
Creole6	Criollo Cubano	CUB	Cuba	50	19	0.793	0.123	0.761	0.080	7.58	2.36	4.92	1.17
Creole6	Siboney	SIB	Cuba	50	19	0.746	0.172	0.762	0.116	8.05	2.30	5.05	1.08
Creole6	Criollo Ecuatoriano	ECU	Ecuador	12	19	0.732	0.174	0.771	0.100	6.63	2.11	5.23	1.22
Creole6	Criollo Chiapas	CHI	Mexico	30	19	0.741	0.145	0.782	0.091	7.84	1.57	5.23	0.89
Creole6	Criollo Pilcomayo	PIL	Paraguay	36	19	0.764	0.125	0.768	0.096	7.53	1.74	5.07	1.02
Iberian1	Alentejana	ALT	Portugal	38	19	0.648	0.121	0.688	0.104	5.79	1.32	4.08	0.81
Iberian1	Arouquesa	ARO	Portugal	70	19	0.717	0.133	0.745	0.106	8.05	2.61	4.78	0.96
Iberian1	Barrosã	BAR	Portugal	69	19	0.697	0.166	0.696	0.149	6.53	1.71	4.27	0.89
Iberian1	Cachena	CAC	Portugal	51	19	0.728	0.157	0.727	0.147	7.68	1.95	4.69	0.90
Iberian1	Garvonesa	GAR	Portugal	39	19	0.677	0.160	0.651	0.146	6.16	1.89	3.97	1.03
Iberian1	Marinhoa	MRI	Portugal	46	19	0.689	0.137	0.692	0.125	6.11	1.73	4.15	0.91
Iberian1	Maronesa	MRO	Portugal	47	19	0.685	0.145	0.693	0.117	6.68	1.63	4.28	0.83
Iberian1	Mertolenga	MER	Portugal	64	19	0.641	0.129	0.722	0.123	7.74	2.45	4.59	0.99
Iberian1	Mirandesa	MIR	Portugal	54	19	0.615	0.126	0.623	0.114	5.63	1.07	3.55	0.63
Iberian1	Preta	PRE	Portugal	60	19	0.653	0.151	0.674	0.156	7.05	2.61	4.26	1.12
Iberian1	Alistana	ALS	Spain	50	19	0.664	0.136	0.711	0.110	7.37	1.67	4.41	0.84
Iberian1	Negra Andaluza	NAN	Spain	21	19	0.634	0.170	0.708	0.117	6.16	2.14	4.45	1.11
Iberian2	Minhota	MIN	Portugal	50	19	0.767	0.121	0.725	0.105	7.68	2.24	4.64	0.84
Iberian2	Ramo Grande	RGD	Portugal	44	19	0.688	0.098	0.721	0.099	7.53	2.39	4.57	0.94
Iberian2	Berrenda en Colorado	BCO	Spain	40	19	0.731	0.098	0.779	0.069	7.68	2.16	5.11	0.99
Iberian2	Berrenda en Negro	BNE	Spain	30	19	0.595	0.196	0.635	0.124	5.21	1.55	3.80	0.96
Iberian2	Bruna de los Pirineos	BRP	Spain	50	19	0.687	0.130	0.697	0.131	7.53	2.09	4.46	1.06
Iberian2	Marismeña	MAR	Spain	50	19	0.724	0.118	0.740	0.096	7.79	2.55	4.67	0.80
Iberian2	Mostrenca	MOS	Spain	50	19	0.602	0.133	0.608	0.117	5.42	1.39	3.57	0.70
Iberian2	Pajuna	PAJ	Spain	38	19	0.711	0.111	0.741	0.094	7.37	2.17	4.69	0.99
Iberian2	Parda de Montaña	PMO	Spain	50	19	0.700	0.100	0.710	0.089	7.47	1.81	4.49	0.85
Iberian2	Serrana de Teruel	STE	Spain	50	19	0.727	0.115	0.762	0.094	7.95	1.99	4.94	1.04
Iberian3	Vaca Canaria	VCA	Spain	50	19	0.729	0.099	0.768	0.071	8.00	2.29	4.92	0.84
Iberian3	Vaca Palmera	VPA	Spain	50	19	0.633	0.184	0.626	0.177	5.58	1.84	3.76	1.10
Iberian4	Brava de Lide	BDL	Portugal	43	19	0.565	0.156	0.631	0.162	5.47	1.81	3.71	1.00
Iberian4	Asturiana de los Valles	ASV	Spain	50	19	0.728	0.139	0.754	0.105	8.63	2.17	5.07	1.17
Iberian4	Asturiana de Montaña	ASM	Spain	50	19	0.674	0.138	0.723	0.108	7.16	2.22	4.57	1.05
Iberian4	Avileña	AVI	Spain	50	19	0.688	0.116	0.731	0.123	7.37	2.43	4.70	1.10
Iberian4	Betizu	BET	Spain	20	19	0.665	0.133	0.730	0.082	5.68	1.45	4.42	0.91
Iberian4	Mallorquina	MAL	Spain	50	19	0.542	0.238	0.577	0.195	4.37	1.54	3.17	0.93
Iberian4	Menorquina	MEN	Spain	50	19	0.636	0.151	0.628	0.136	5.84	1.98	3.78	0.98
Iberian4	Monchina	MON	Spain	50	19	0.732	0.121	0.751	0.087	8.11	2.28	4.88	0.99
Iberian4	Morucha	MOR	Spain	50	19	0.704	0.088	0.754	0.086	7.74	1.76	4.81	0.82
Iberian4	Pasiega	PAS	Spain	50	19	0.686	0.129	0.721	0.115	7.53	2.20	4.67	1.02
Iberian4	Pirenaica	PIR	Spain	50	19	0.704	0.095	0.725	0.083	7.16	2.06	4.57	0.87
Iberian4	Retinta	RET	Spain	50	19	0.736	0.089	0.758	0.095	7.53	2.61	4.83	1.03
Iberian4	Rubia Galega	RGA	Spain	50	19	0.696	0.148	0.713	0.139	7.47	1.95	4.57	1.08
Iberian4	Sayagesa	SAY	Spain	50	19	0.686	0.105	0.743	0.094	7.32	1.60	4.73	0.84
Iberian4	Toro de Lidia	TDL	Spain	50	19	0.587	0.140	0.651	0.149	6.32	2.11	4.03	1.08
Iberian4	Tudanca	TUD	Spain	50	19	0.659	0.140	0.696	0.129	7.16	2.34	4.50	1.27
British	Aberdeen Angus	AAN	Argentina;USA	62	19	0.633	0.157	0.682	0.149	6.26	1.88	4.12	0.87
British	British White Cattle	BWC	USA	19	19	0.667	0.117	0.676	0.122	5.00	1.29	4.01	0.96
British	Hereford	HER	Argentina;Mexico;USA	88	19	0.655	0.085	0.704	0.078	6.63	1.54	4.14	0.87
British	Jersey	JER	USA	20	19	0.666	0.157	0.648	0.112	4.79	1.03	3.63	0.64
British	Shorthorn	SHR	USA	11	19	0.690	0.189	0.656	0.144	5.16	1.98	4.23	1.36
Continental European	Brown Swiss	BSW	Mexico	29	19	0.739	0.100	0.726	0.103	6.79	2.15	4.61	1.08
Continental European	Charolais	CHA	Portugal	58	19	0.682	0.156	0.705	0.141	6.95	1.99	4.45	1.13
Continental European	Friesian	FRI	Portugal	50	19	0.732	0.113	0.714	0.109	6.89	2.40	4.45	1.12
Continental European	Limousin	LIM	Portugal	47	19	0.740	0.074	0.743	0.079	6.95	1.99	4.65	0.93
Indicine	Guzerat	GUZ	Brazil	15	19	0.606	0.142	0.643	0.120	5.05	1.61	3.90	1.02
Indicine	Nelore	NEL	Brazil	28	19	0.544	0.191	0.603	0.122	5.53	1.58	3.70	0.85
Indicine	Sindi	SIN	Brazil	11	19	0.651	0.190	0.672	0.131	5.42	1.77	4.43	1.22
Indicine	Gyr	GYR	Brazil/Mexico	23	19	0.607	0.185	0.658	0.135	5.79	1.65	4.13	0.94
Indicine	Cuban Zebu	CZE	Cuba	50	19	0.713	0.202	0.710	0.179	7.53	2.06	4.73	1.22
Indicine	Brahman	BRH	USA	41	19	0.682	0.132	0.699	0.106	7.74	2.40	4.51	0.99
**Totals**		**82**		**3383**									
