# Supplementary figures and images for: Analysis of conservation priorities of Iberoamerican cattle based on autosomal microsatellite markers
Source: Genet Sel Evol. 2013 Sep 30;45(1):35. doi: 10.1186/1297-9686-45-35 (PMC3851275; doi:10.1186/1297-9686-45-35)

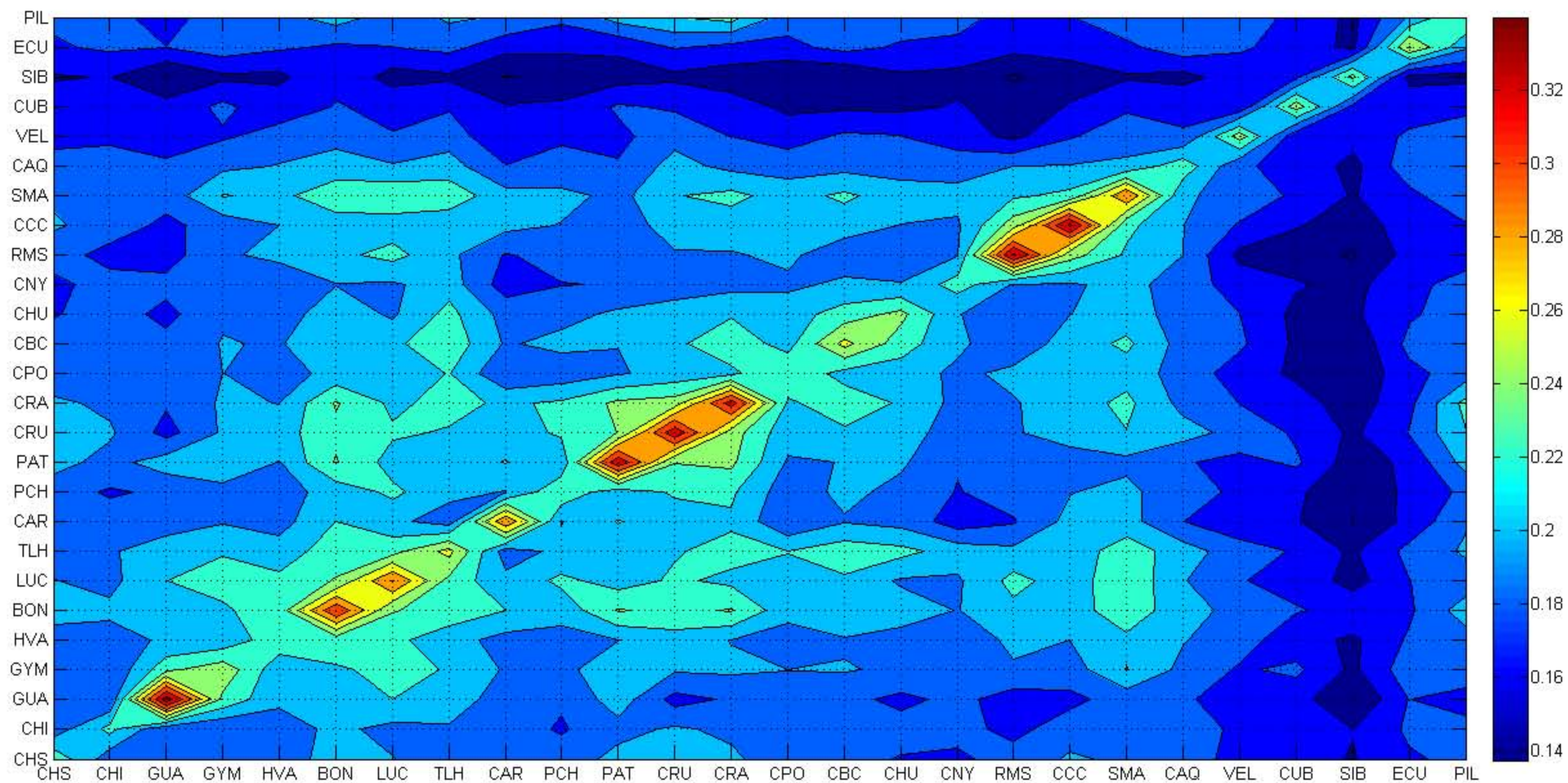

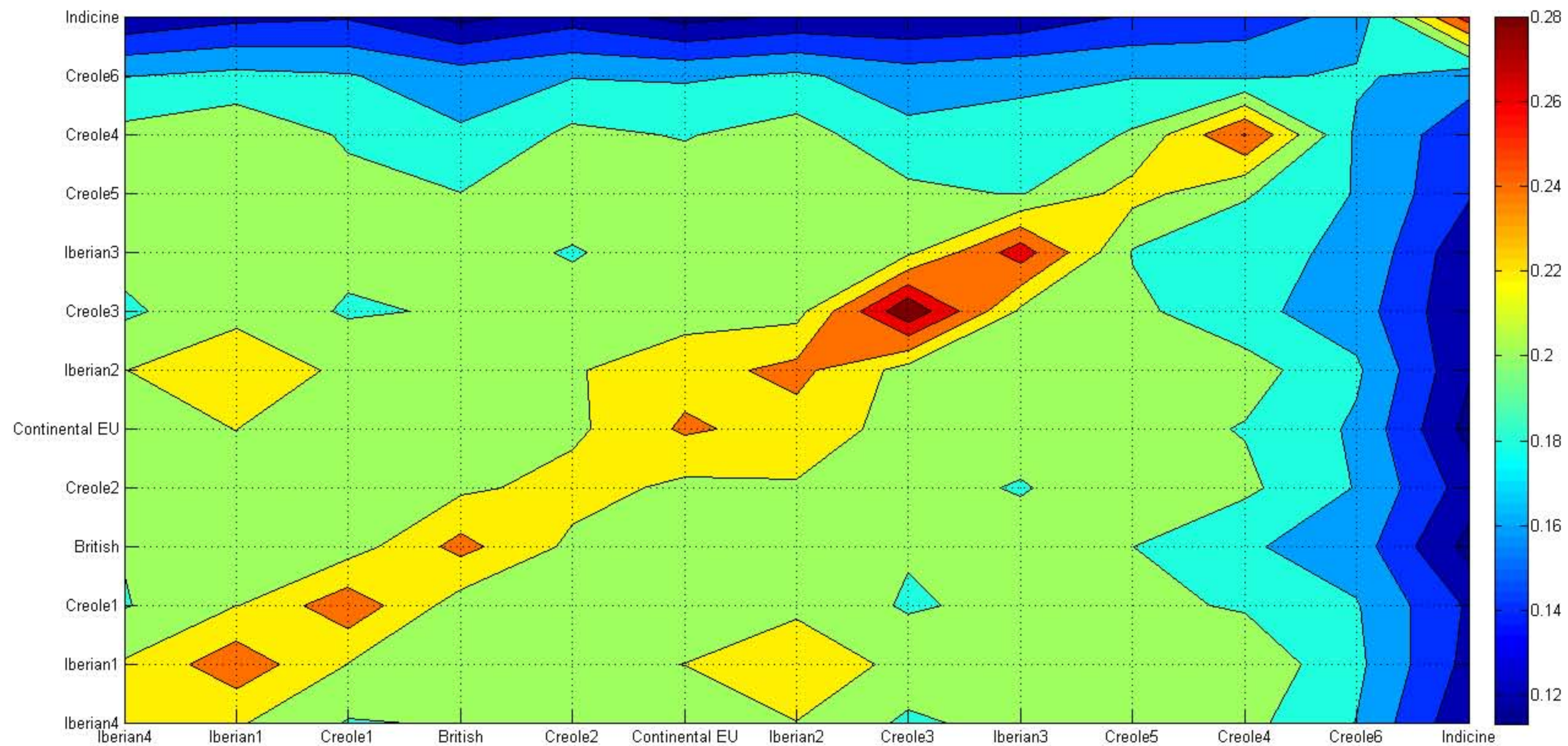

Supplement: Additional file 2: Figure S1 — Contour plots of average coancestries (fm). Description: Creole cattle breeds (a) and geographical breed groups (b) were sorted according to the respective kinship phylogenies. Breed acronyms are defined as follows: CRA, Cr. Argentino; PAT, Cr. Patagonico; CAR, Caracú; CRU, Cr. Uruguayo; BON, Blanco Orejinegro; HVA, Hartón del Valle; LUC, Lucerna; PCH, Pampa Chaqueño; CCC, Costeño con Cuernos; RMS, Romosinuano; GUA, Guabalá; GUY, Guaymí; SMA, Sanmartinero; CBC, Cr. Baja California; CHU, Cr. Chihuahua; CNY, Cr. Nayarit; CPO, Cr. Poblano; TLH, Texas Longhorn; CAQ, Caqueteño; CHS, Chino Santandereano; CAS, Cr. Casanareño; VEL, Velasquez; CUB, Cr. Cubano; SIB, Siboney; ECU, Cr. Ecuatoriano; CHI, Cr. Chiapas; PIL, Cr. Pilcomayo. [file 1297-9686-45-35-S2.pdf]

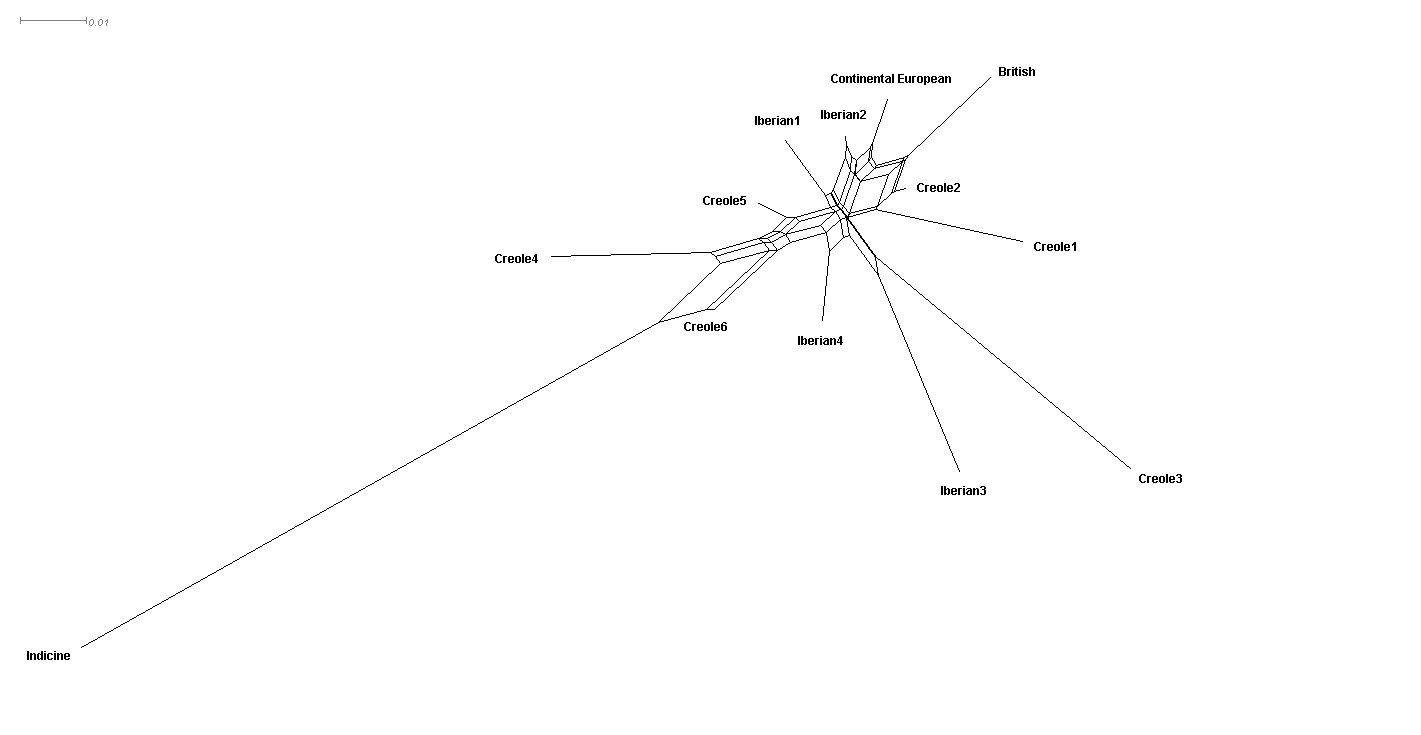

Supplement: Additional file 3: Figure S2 — Description: Neighbor-net graph of Reynolds distances showing the genetic relationships among the 13 geographical breed groups studied. [file 1297-9686-45-35-S3.png]

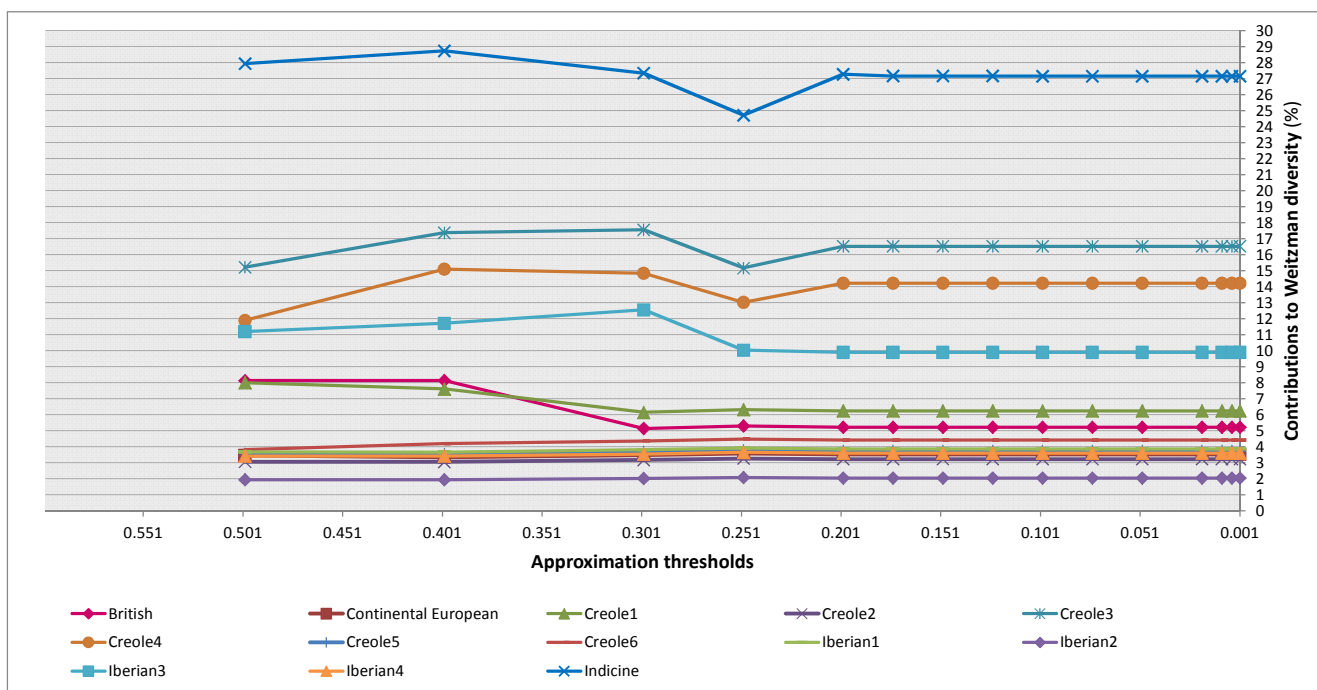

Supplement: Additional file 4: Figure S3 — Variation of breed-group contributions to Weitzman diversity using the approximation algorithm of Garcia et al. [22]. Description: This figure shows that the approximate estimates of the Weitzman diversity and exact methods are in full agreement only for thresholds between 0.300 and 0.200. Given the large number of breeds involved, applying the approximate procedure would be too computationally demanding, thus geographical breed groups were defined for the analysis of conservation priorities of worldwide cattle. [file 1297-9686-45-35-S4.pdf]
